# The Correlation Between Potential “Anti- Cancer” Trace Elements and the Risk of Breast Cancer: A Case-Control Study in a Chinese Population

**DOI:** 10.3389/fonc.2021.646534

**Published:** 2021-08-10

**Authors:** Heng Xue, Rui Qiao, Lailai Yan, Siyu Yang, Yongming Liang, Yaqiong Liu, Qing Xie, Ligang Cui, Bing Cao

**Affiliations:** ^1^Department of Ultrasound, Peking University Third Hospital, Beijing, China; ^2^Baotou Medical College, Inner Mongolia University of Science and Technology, Baotou, China; ^3^Department of Laboratorial Science and Technology, School of Public Health, Peking University, Beijing, China; ^4^Vaccine Research Center, School of Public Health, Peking University, Beijing, China; ^5^Tianjin Center for Disease Control and Prevention, Tianjin, China; ^6^Department of Laboratory Medicine, Peking University Third Hospital, Beijing, China; ^7^Key Laboratory of Cognition and Personality (SWU), Faculty of Psychology, Ministry of Education, Southwest University, Chongqing, China

**Keywords:** selenium, strontium, anti-cancer, malignant, ICP-MS

## Abstract

**Backgrounds:**

Breast cancer is a heterogeneous disease without clear pathogenesis and effective primary prevention. The “anti-cancer” effects of several trace elements have received increasing attention in recent years. The main purpose of current study is to explore the differences of three potential “anti-cancer” trace elements selenium (Se), molybdenum (Mo), and strontium (Sr) between patients with malignant breast tumors and healthy controls.

**Methods:**

We conducted a case–control study in 45 patients with malignant breast tumors as cases and 95 healthy volunteers as controls from Peking University Third Hospital, Beijing, China. The serum concentrations of trace elements were evaluated by using inductively coupled plasma mass spectrometry (ICP-MS).

**Results:**

The cases may have a lower Se levels when compared with controls (cases: 106.22 ng/ml, SD: 20.95 ng/ml; controls: 117.02 ng/ml, IQR: 22.79 ng/ml, p = 0.014). High levels of Se were a protective factor from breast cancer after adjusting the potential confounders of age, BMI, smoking, drinking, and menopause status (OR = 0.395, 95% CI, 0.178, 0.877, *p* = 0.023). The levels of Sr were lower in cases with high histologic grade when compared to low histologic grade (low histologic grade: 49.83 ng/ml, IQR: 41.35–62.60 ng/ml; high histologic grade: 40.19 ng/ml, IQR: 39.24–47.16 ng/ml, *p* < 0.05).

**Conclusions:**

Our findings herein supported that Se has protective effects to avoid malignant breast tumors and Sr has protective effects to avoid poorly differentiated malignant breast tumors. Exploring “anti-cancer” related trace elements and their associations with breast cancer will assist for the early prevention and intervention for the disease.

## Introduction

Breast cancer is one of the topmost prevalent malignancies and the major cause of cancer-related death in women worldwide (1, 2). It is a heterogeneous disease without clear pathogenesis and effective primary prevention (3). The incidence of breast cancer has steadily increased in the past years. With the improvements in early detection and treatment achieved, most of the women are diagnosed in the early stage of breast cancer (4). The incidence of breast cancer is generally considered to be the interaction of environmental factors and genetic factors, and approximately 5–10% of breast cancers are inheritable (5). The occurrence and development of breast cancer are related to a variety of physiological factors, including but not limited to age, menstrual status, fertility, breastfeeding, and obesity (6). Accumulating clinical studies and animal experiments have shown that the incidence of breast cancer is related to the long-term exposure to high levels of environmental estrogen and oxidative stress (7, 8). Recent studies reported that some metal elements can activate estrogen receptors by mimicking the action of physiological estrogen to accelerate the development of breast cancer (9).

The association between trace element exposure and breast cancer has received increasing attention in recent years. There have been a large number of researches assessing the association between breast cancer and essential trace elements (9, 10). With the continuous deepening of research on trace elements, more and more researches link trace elements with cancer. Some trace elements with relatively low concentrations in human body have shown obvious anti-cancer and anti-oxidant effects in previous studies. For example, selenium (Se) and molybdenum (Mo) have been proved to be involved in reactive oxygen species generation and to play crucial role in functioning of antioxidant enzymes (11, 12). However, significant higher levels of Mo in serum were found in breast cancer patients than controls in a Korean population (13). Se-containing molecules that exert antioxidant properties and modulate targets are associated with tumor growth, metastasis, angiogenesis, and drug resistance. It also participates in processes related to carcinogenesis, such as inhibition of tumor formation and regression. Strontium (Sr), which is an alkali earth metal, has similar chemical characteristics with calcium. It is present in all living organisms and can be superiorly absorbed by bones (14), and Sr-containing molecules have been used to kill tumor cells and induce tumor cell apoptosis in patients with breast cancer (15).

With the development and progress of detection technology, the exploration of the functions of trace elements is not limited to the elements we know well (e.g., calcium, iron, and zinc), and the exploration of relatively low trace elements has gradually increased. To the best of our knowledge, only a few studies explored the association between potential “anti-cancer” trace elements and breast cancer, especially in Chinese population. The inductively coupled plasma mass spectrometry (ICP-MS) can be used to quantify several of the medically interesting trace elements precisely and accurately simultaneously (16). Meanwhile, the serum sample can represent the circulating levels of trace elements, which is suitable for the determination of most of the essential and toxic elements (17). Based on the search of previous literature, our current study aimed to explore the differences of three potential “anti-cancer” trace elements (i.e., Se, Mo, and Sr) in serum samples between patients with malignant breast tumors and healthy controls using an ICP-MS-based analysis. Meanwhile, the associations between the histological grade of malignant tumors and three trace elements were also assessed.

## Methods

### Study Design and Participants

For this case–control study, all participants were recruited from Peking University Third Hospital in Beijing, China during the period of time between 2017 and 2018. During the study period, we recruited 45 female inpatients who met the histopathological diagnosis of malignant breast disease and equal to or more than 18 years of age as case group. The malignant breast disease was obtained from definite pathological histological diagnosis based on ultrasound-guided breast biopsy. Ninety-five healthy volunteers, participating in the annual health examinations, were included as control group based on the following criteria: (1) their age matched with the case group, and (2) they were not diagnosed with breast diseases ever. All control groups were recruited at the same time and from the same district as case group. We excluded (1) the pregnant and postpartum women; (2) the female who have been diagnosed with severe physical disease, liver, kidney, or endocrine diseases, or any other severe or unstable medical illness; (3) the women who have history of occupational metal exposure, such as mental manufacturing industries; and (4) the women who have recent infection. All participants provided written or verbal informed consent to have their samples and information collected and used for this study. The scoring of histological grade of malignant tumors was according to glandular tubules formation (1–3 points), the shape and size of nucleus and irregular chromatin (1–3 points), and chromatin and mitotic phase (1–3 points). The malignant tumors with a total score of 3–5 points were designated as grade I, 6–7 points were designated as grade II, and 8–9 points were designated as grade III. All the inpatients in case group received a standard diet provided by the hospital prior to collecting the serum samples. The living environment of them was similar. The fasting blood samples were collected from cases before anti-cancer treatment following overnight fasting. Thus, it can partly eliminate the interference of diet and environment.

The research protocol was approved by the research ethics committee of the Peking University Third Hospital in Beijing, China (IRB-2018-413-02), and all methods were carried out in accordance with the approved guidelines.

### Serum Sample Preparation and Trace Element Analysis

Serum samples of case group were collected before pathological diagnosis. At that time, the participants did not receive chemotherapy or other anti-cancer therapy. Approximately 5 ml fasting blood vein was collected from all participants (12 h fasting, collected between 7 and 9 a.m.), centrifuged at 3000 rpm for 5 min to obtain serum. Then, the serum aliquot was transferred into Eppendorf tubes and stored at -80°C until use. The serum samples of the control group were collected with the same protocols as the case group. The detection of Se, Mo, and Sr was conducted by using ICP-MS (7700x, Agilent, USA) after the following pretreatment: 0.1 ml of each serum sample was transferred to a quartz tube, added 0.1 ml mixed internal standard elements (indium: 2 ng/ml, rhodium: 20 ng/ml), then added 1.8 ml 1% nitric acid and mixed (18). The selected isotopes of the three trace elements are as follows: ^95^Mo, ^78^Se, and ^88^Sr. The instrument parameters of ICP-MS are as follows: gas flow (1.0 L/min), helium flow (4.5 ml/min), power of radio frequency generator (1.50 KW), and integration time (300 ms). The detailed main parameters of ICP-MS are shown in **Supplementary Table 1**. As shown in **Supplementary Table 2**, all measured metal concentrations were included in the range of their certified reference material (CRM).

### Statistical Analysis

All the data analysis was performed using Stata (version 15.0, Stata Corp LP, College Station, TX, USA). For the concentrations of trace elements in the investigated samples, mean and standard deviation (SD) or median and interquartile range (IQR) were computed. Parameter tests or nonparametric statistical tests were used for analyzing the results (independent t-test, Mann–Whitney U test). The median values of the Se, Mo, and Sr concentrations in controls were used as the cut-off value in the logistic regression analysis. Odds ratios (ORs) and their 95% confidence intervals (CIs) were estimated using maximum likelihood methods. The correlations between concentrations of trace elements and histological grade were analyzed with Spearman correlation model. A two-sided p value of less than 0.05 was considered significant.

## Results

### Demographic and Clinical Characteristics of Recruited Participants

The distribution characteristics of the recruited participants were briefly presented in **Table 1**. The control group was easier to collect than case group in the actual situation. In total, 45 patients with malignant breast tumors and 95 healthy controls were included in the analysis. The mean age of cases and controls was 52.69 years (SD = 14.15 years) and 46.96 years (SD = 12.35 years), respectively. It was observed that there were significant differences in smoking, drinking, and menopause status between cases and controls (all *p* > 0.05). The cases (24.12 ± 2.78 kg/m^2^) have higher body mass index (BMI) than controls (23.02 ± 3.21 kg/m^2^). Three of cases (6.7%) have the family history of breast cancer, and none of the controls have the family history of cancer (*p* = 0.011); 37.8% of the cases were overweight and 8.9% were obese.

**Table 1:** Demographic and clinical characteristics of recruited participants.

Variable	Case (n = 45)	Control (n = 95)	p-values
Age, year, mean ± SD	52.69 ± 14.15	46.96 ± 12.35	0.028*^a^*
Smoker (n, %)	1 (2.2)	3 (3.2)	1.000*^b^*
Drinker (n,%)	5 (11.1)	15 (15.8)	0.460*^c^*
Histologic grade (n, %)			
I	6 (13.3)		
II	24 (53.3)		
III	15 (33.3)		
Menopause status (n, %)			
Yes	25 (55.6)	42 (44.2)	0.209*^c^*
No	20 (37.3)	53 (55.8)	
Family history of cancer (n, %)			
Yes	3 (6.7)	0 (0.0)	0.011*^b^*
No	42 (93.3)	95 (100.0)	
BMI, kg/m^2^, mean ± SD	24.12 ± 2.78	23.02 ± 3.21	0.049*^a^*
Underweight	0 (0.0)	3 (3.2)	
Normal	24 (53.3)	59 (62.1)	
Overweight	17 (37.8)	25 (26.3)	
Obesity	4 (8.9)	8 (8.4)	

BMI, body mass index.

a p values were calculated by two-tailed independent t-test.

b Calculated using the Fisher’s exact test.

c Calculated using the Pearson’s chi-square test.

### Comparisons of Concentrations of Se, Mo, and Sr Between Cases and Controls

Mann–Whitney U tests were performed to analyze the differences of Mo and Sr levels between case group and control group, while the independent t test was used to compare the differences of Se levels between the two groups. The results showed that no significant differences existed in the levels of Mo and Sr between two groups (*p* > 0.05). The levels of Se in cases were significantly lower than those in control group (cases: 106.22 ng/ml, SD: 20.95 ng/ml; controls: 117.02 ng/ml, IQR: 22.79 ng/ml, *p* = 0.014). The comparisons of concentrations of Se, Mo, and Sr between cases and controls are presented in **Table 2**.

**Table 2:** Comparisons of concentrations for Se, Mo and Sr between cases and controls.

ETMs	Unit	Case (n = 45)	Control (n = 95)	*p*-values
Se	ng/ml, mean ± SD	106.22 ± 20.95	117.02 ± 22.79	0.014*^a^*
Mo	ng/ml, median (IQR)	1.84 (1.57, 2.09)	1.88 (1.62, 2.26)	0.733*^b^*
Sr	ng/ml, median (IQR)	44.53 (39.85, 54.60)	46.64 (40.57, 55.43)	0.628*^b^*

a Calculated by two-tailed independent t-test.

b Calculated by two-tailed Mann–Whitney U-tests.

Based on the cut-off values of the median value of the trace element levels in controls, the results of logistic regression are shown in **Table 3**. Univariate analyses revealed that significant difference existed between the case group and control group for Se (*p* < 0.05). Significance was maintained after adjusting the potential confounders of age, BMI, smoking, drinking, and menopause status (Model 3: AOR = 0.395, 95% CI: 0.178, 0.877, *p* = 0.023). We did not find any association between cases and the other two trace elements before and after adjusting potential confounders (all *p* > 0.05).

**Table 3:** Odds ratios (ORs) of Se, Mo, and Sr of serum dichotomized by the corresponding medians of controls.

Variable	Group	Case	Control	Model 1		Model 2		Model 3	
				UOR (95%CI)	p	AOR1 (95%CI)	p	AOR2 (95%CI)	p
**Se**	Low	32 (71.1)	48 (50.5)	0.433 (0.202, 0.925)	0.031	0.403 (0.182, 0.889)	0.024	0.395 (0.178, 0.877)	0.023
	High	13 (28.9)	47 (49.5)						
**Mo**	Low	26 (57.8)	48 (50.5)	0.783 (0.384, 1.597)	0.502	0.712 (0.338, 1.5)	0.372	0.648 (0.3, 1.4)	0.270
	High	19 (42.2)	47 (49.5)						
**Sr**	Low	25 (55.5)	48 (50.5)	0.778 (0.381, 1.592)	0.493	0.636 (0.297, 1.361)	0.244	0.622 (0.286, 1.349)	0.229
	High	20 (44.5)	47 (49.5)						

UOR, Unadjusted odds ratio; AOR, adjusted odds ratio.

Model 1: Calculated by an unconditional logistic regression model.

Model 2: Calculated by an unconditional logistic regression model with adjustment of age and BMI.

Model 3: Calculated by an unconditional logistic regression model with adjustment of model 2 plus smoking, drinking, and menopause status.

### Comparisons of Concentrations for Se, Mo, and Sr Between Cases With Low and High Histologic Grade

**Figure 1:**
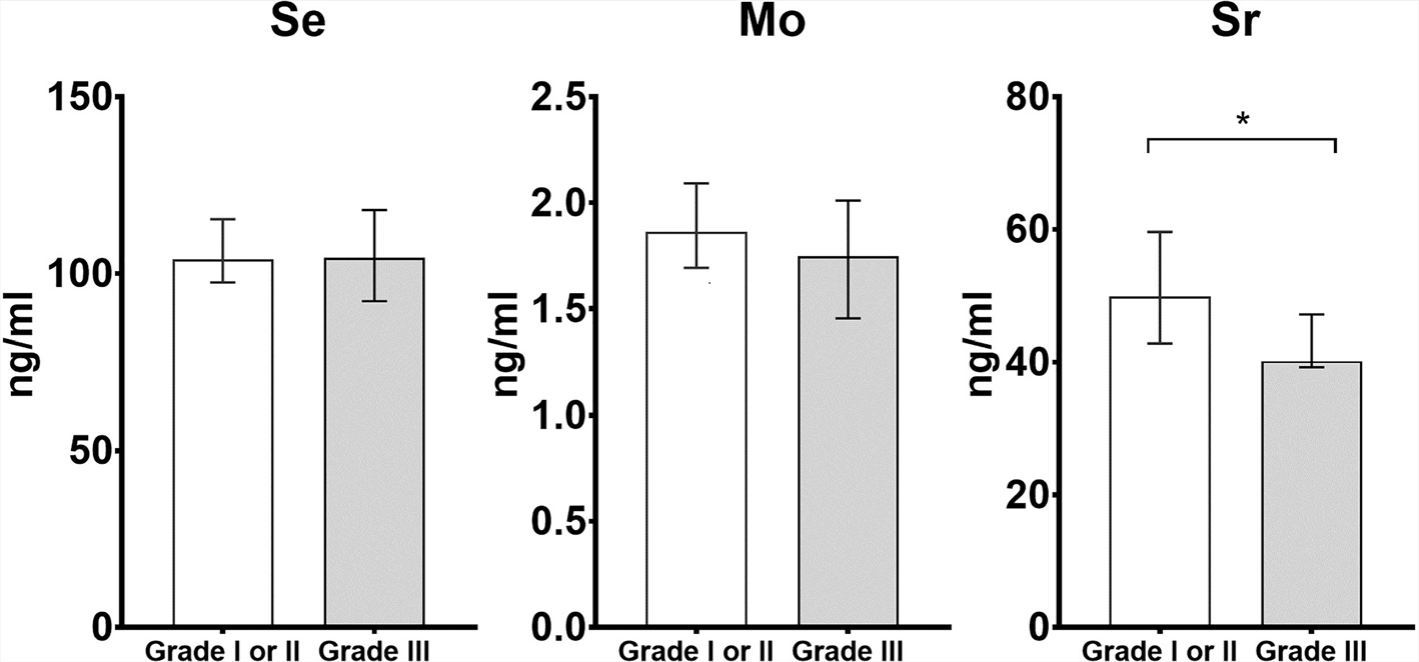
Histograms of differential trace elements (i.e., Se, Mo, and Sr) for the comparison between cases with low histologic grade (i.e., Grade I or II) and high histologic grade (i.e., Grade III). The x-axis is the histologic grade, and the y-axis is the concentration of Se, Mo and Sr (ng/ml). * statistically significant with *p* < 0.05 in the comparison between cases with low and high histologic grade.

We also conducted the analysis for comparing the concentrations of Se, Mo, and Sr between cases with low and high histologic grade. The cases with histologic grade of I and II were defined as low histologic grade (n = 30), while with histologic grade III were defined as high histologic grade (n = 15). The results of comparisons are shown in **Figure 1**. The levels of Sr were lower in cases with high histologic grade when compared to low histologic grade (low histologic grade: 49.83 ng/ml, IQR: 41.35–62.60 ng/ml; high histologic grade: 40.19 ng/ml, IQR: 39.24–47.16 ng/ml, *p* < 0.05).

## Discussion

Our case–control study evaluated the associations between malignant breast tumors and three previously defined potential “anti-cancer” trace elements Se, Mo, and Sr from a Chinese population. The main findings of our study are that the cases may have a lower Se levels when compared with controls, and the levels of Sr were lower in cases with high histologic grade when compared to low histologic grade. Our findings herein supported that Se has protective effects to avoid malignant breast tumors and Sr has protective effects to avoid poorly differentiated malignant breast tumors.

Selenium is an important composition of antioxidant enzyme glutathione peroxidase, which could inhibit the synthesis of tumor cell protein and DNA. Se-containing molecules exert antioxidant properties and participate in maintaining equilibrium of a healthy organism (19). Epidemiological and preclinical evidence illustrated that Se is a trace element with anti-cancer activity and is associated with tumor growth, metastasis, angiogenesis, and drug resistance (20, 21). Accumulating evidence supported that Se has protective effects of breast cancer and the serum levels of Se can predict survival after breast cancer (13, 22–24). A case–control study from Malaysia reported that the risk of breast cancer decreased with the increasing of selenium intake (25). Similar with our findings, Charalabopoulos et al. reported a strong negative correlation between serum concentrations of Se and the risk of breast cancer, while there was no correlation between serum/tissue Se concentration and stage of the disease (26). Although we did not find the significant association between histologic grade and Se, previous studies have pointed out that serum Se concentrations decrease with the poorly differentiated malignant breast tumors (27). Further studies are needed to expand the sample size of each histologic grade group to explore the association between Se levels and different differentiation and severity of malignant tumors.

The role of Sr in the metabolism of human body has not well been studied as yet. We observed lower concentrations of Sr in cases with high histological grade compared to those with low histological grade. Strontium is an alkali earth metal with similar chemical behavior to calcium, and has been reported to interfere with absorption and metabolism of calcium (28). Inconsistent with our findings herein, a previous case–control study indicated that higher urinary concentrations of strontium were associated with potential breast cancer, suggesting that Sr plays a role in the development of the disease (29). Meanwhile, the radioactive Sr has been reported to have the potential effects of anti-cancer and has been used for the treatment of multiple bone metastases of breast cancer (30, 31). Nevertheless, our current data provide a basis for future research on the role of Sr in the metabolic pathway of breast cancer, especially for the protective effects to avoid the poorly differentiated malignant breast tumors.

Molybdenum is an important component of enzymes, such as xanthine oxidase, oxidase, nicotinate hydroxylase, and sulfite oxidase (32). Many studies have shown that Mo is involved in ROS generation and is essential for cell metabolism (33). The deficiency of Mo can be correlated to the development of esophageal cancer (34). However, a previous study reported that high Mo might induce hepatocyte apoptosis through a mitochondrial pathway (35). Our study did not find association between Mo and breast cancer. The potential anti-cancer effects of Mo on breast cancer should be cautiously considered (36).

The current study provided valuable clues for exploring the potential “anti-cancer” effects of the trace elements Se, Mo, and Sr on breast cancer. Several limitations in our study must be addressed. Firstly, we cannot confirm the actual causality between malignant breast tumors and changes of trace elements Se, Mo, and Sr from an observational study. Secondly, all subjects were from Peking University Third Hospital in Beijing, which is not sufficiently representative of the whole population and may lead to selection bias. Thirdly, there are relatively few cases with each histologic grade group, and we merged Grade I and Grade II in our analysis. Moreover, the levels of trace elements in serum may be disturbed by the body physiological status, current nutrition, and exogenous environment. Although all cases received a standard diet provided by the hospital prior to collecting the serum samples, the living environment of them was similar; all fasting blood samples of cases were collected before anti-cancer treatment following overnight fasting, the influence of dietary and environmental factors cannot be fully eliminated, and our established methods and findings in this work should be extrapolated with caution. Despite the above limitations, the reported results in the current study provide rationale to explore the association between breast cancer and the trace elements Se, Mo, and Sr, which encourages researchers to perform further longitudinal studies to explore the “anti-cancer” effects of these elements on preventing the prevalence of breast cancer. The effects of trace elements Se, Mo, and Sr in tumor tissue and tumor cells, and their molecular metabolism in breast cancer also should be considered in future researches.

## Conclusion

Our current case–control study illustrated that Se levels were lower in patients with malignant breast tumors than in controls. Lower levels of Sr were associated with high histologic grade in cases. These findings provide new basis for further research regarding the mechanism of Se and Sr for occurrence and development of breast cancer. Specifically, exploration with “anti-cancer” related trace elements and their association with breast cancer will assist in the early prevention and intervention for the disease.

## Data Availability Statement

The raw data supporting the conclusions of this article will be made available by the authors, without undue reservation.

## Ethics Statement

The studies involving human participants were reviewed and approved by research ethics committee of the Peking University Third Hospital. The patients/participants provided their written informed consent to participate in this study.

## Author Contributions

BC, LC, and RQ conceived and designed the study. HX, SY, YQL, QX, and LY collected the data and performed the statistical analysis. BC, YML, and LY contributed to the discussion. RQ, LY, SY, BC and LC revised the paper. All authors contributed to the article and approved the submitted version.

## Funding

This work was sponsored by the Research Startup Fund of Southwest University (SWU019039). The supporting foundation had no involvement in study design, the collection, analysis, or interpretation of data, writing of the report; or in the decision to submit the article for publication.

## Conflict of Interest

The authors declare that the research was conducted in the absence of any commercial or financial relationships that could be construed as a potential conflict of interest.

## Publisher’s Note

All claims expressed in this article are solely those of the authors and do not necessarily represent those of their affiliated organizations, or those of the publisher, the editors and the reviewers. Any product that may be evaluated in this article, or claim that may be made by its manufacturer, is not guaranteed or endorsed by the publisher.
