# Supplementary material for: The Correlation Between Potential “Anti- Cancer” Trace Elements and the Risk of Breast Cancer: A Case-Control Study in a Chinese Population
Source: Front Oncol. 2021 Aug 10;11:646534. doi: 10.3389/fonc.2021.646534 (PMC8383177; doi:10.3389/fonc.2021.646534)
Supplement: Supplementary file 1 [file Table_1.docx]

**Supplemental Table 1. The main parameters of ICP-MS (**7700x**)**

| **Variables** | **Parameters** |
| --- | --- |
| Gas flow | 1.0 L/min |
| Helium flow | 4.5 mL/min |
| power of radio frequency generator | 1.50 KW |
| Integration time | 300 ms |
| Scanning | 3 points |
