# Supplementary material for: The Correlation Between Potential “Anti- Cancer” Trace Elements and the Risk of Breast Cancer: A Case-Control Study in a Chinese Population
Source: Front Oncol. 2021 Aug 10;11:646534. doi: 10.3389/fonc.2021.646534 (PMC8383177; doi:10.3389/fonc.2021.646534)
Supplement: Supplementary file 2 [file Table_2.docx]

**Supplemental Table 2. Measured and reference concentration of certified reference material (CRM)**

| **Elements** | **mean value（ n=3）** | **Reference value** | **CRM matrix** | **Manufacturer** |
| --- | --- | --- | --- | --- |
| **Se** | 110.2 ng/mL | 103 (82.4-124) ng/mL | Serum | RECIPE Chemicals + Instruments GmbH |
| **Mo** | 1.93 ng/mL | 1.82 (1.37-2.28) ng/mL | Plasma | RECIPE Chemicals + Instruments GmbH |
| **Sr** | 8.62 μg/g | 8.17 (7.48-8.86) μg/g | Hair | Shanghai Institute of Applied Physics, Chinese Academy of Sciences |
